# Direct LAMP Assay without Prior DNA Purification for Sex Determination of Papaya

**DOI:** 10.3390/ijms17101630

**Published:** 2016-09-24

**Authors:** Chi-Chu Tsai, Huei-Chuan Shih, Ya-Zhu Ko, Ren-Huang Wang, Shu-Ju Li, Yu-Chung Chiang

**Affiliations:** 1 Kaohsiung District Agricultural Research and Extension Station, Pingtung 908, Taiwan; tsaicc@mail.kdais.gov.tw (C.-C.T.); rhwang@mail.kdais.gov.tw (R.-H.W.); gigi7366kimo@yahoo.com.tw (S.-J.L.); 2 Department of Biological Science and Technology, National Pingtung University of Science and Technology, Pingtung 912, Taiwan; 3 Department of Nursing, Meiho University, Pingtung 912, Taiwan; x00002213@meiho.edu.tw; 4 Department of Biological Sciences, National Sun Yat-sen University, Kaohsiung 804, Taiwan; abscl77512@hotmail.com; 5 Department of Biomedical Science and Environment Biology, Kaohsiung Medical University, Kaohsiung 807, Taiwan

**Keywords:** papaya, sex determination, loop-mediated isothermal amplification (LAMP), without DNA purification

## Abstract

Papaya (*Carica papaya* L.) is an economically important tropical fruit tree with hermaphrodite, male and female sex types. Hermaphroditic plants are the major type used for papaya production because their fruits have more commercial advantages than those of female plants. Sex determination of the seedlings, or during the early growth stages, is very important for the papaya seedling industry. Thus far, the only method for determining the sex type of a papaya at the seedling stage has been DNA analysis. In this study, a molecular technique—based on DNA analysis—was developed for detecting male-hermaphrodite-specific markers to examine the papaya’s sex type. This method is based on the loop-mediated isothermal amplification (LAMP) and does not require prior DNA purification. The results show that the method is an easy, efficient, and inexpensive way to determine a papaya’s sex. This is the first report on the LAMP assay, using intact plant materials-without DNA purification-as samples for the analysis of sex determination of papaya. We found that using high-efficiency DNA polymerase was essential for successful DNA amplification, using trace intact plant material as a template DNA source.

## 1. Introduction

Papaya (*Carica papaya* L.) is a species native to tropical America and a member of the Caricaceae family [1]. The plant’s three sex types are hermaphrodite, male, and female. Fruits from the female plants have more seeds and less flesh than hermaphrodite plants [2], so they usually have less economic value in the papaya industry [2]. Thus, selecting hermaphrodite plants to cultivate is very important in papaya production. However, sex type cannot be identified visually before flowering because there is no morphological feature that can be used as an indicator in the vegetative stage [3]. Thus, farmers generally grow all three types in one location until flowering time, when papaya sex can be identified.

After sex determination, only the hermaphrodite plant is retained for continued growth. However, this cultivation model wastes a large amount of labor and money. Developing a molecular identification tool [4,5,6] for seedlings would be useful for sex determination. Many studies have been conducted to develop DNA detection systems for the sex determination of papaya. For example, several male-hermaphrodite-specific DNA markers (SCAR markers) were derived from random amplified polymorphic DNA (RAPD) [3,7,8] and amplified fragment length polymorphism (AFLP) [9]. These SCAR markers have been successfully applied to papaya production [10]. Although PCR-based SCAR markers have been used to identify the sex type of papaya plants, they still have several disadvantages; including the high cost of equipment, the time-consuming process, and unavailable field screening [11].

Recently, Notomi et al. developed an easy, efficient, sensitive, and inexpensive DNA analysis tool called loop-mediated isothermal amplification (LAMP) [12]. LAMP and reverse transcription (RT)-LAMP analyses have been largely applied to DNA and RNA detection for various organisms; such as bacteria, parasites, viruses, plants, and animals [12,13,14,15,16,17]. The sex type of papaya is controlled by a male-specific region located on the primitive sex chromosome (the Y chromosome) [18,19]. LAMP analysis has been applied to identify the sex type of papaya, based on the detection of this region [16].

DNA extraction is usually a costly and time-consuming process for enzyme-based genotyping of both animals and plants; particularly in plants that have high contents of polysaccharides, polyphenols, or other secondary metabolites [20,21,22]. Because both DNA polymerase and restriction enzymes are sensitive to DNA quality, clean extracted DNA is generally needed [23,24]. Several studies have developed high-quality DNA purification methods for various plants [25,26,27,28,29,30]. DNA extraction is usually the limiting step for reducing the cost and time needed in molecular assays [31,32]. PCR without DNA purification cannot be done because of the chlorophyll, polysaccharides, and starches in the plant cells; which can inhibit the enzyme reaction [31,32,33,34,35,36,37]. Therefore, many efforts have been made towards the development of new methods for DNA analysis without DNA purification. Several studies on DNA analysis by PCR without DNA purification have been reported using animal cells [33,34,35,36]. A method has also been developed to directly detect viruses infecting the *Phalaenopsis* orchid, using fresh leaves in RT-LAMP analysis [38]. However, a method is still needed to detect markers from intact plant materials-without DNA purification-for the sex determination of papaya using the LAMP assay.

In this study, a LAMP analysis-using intact plant materials as the DNA source-was conducted to identify the gender of papaya. To our knowledge, this is the first report of the LAMP assay being used on intact plant materials for sex determination in papaya. The sensitivity and transferability of this technique to other papaya varieties were also examined. Our results indicate that the DNA screening method is powerful, efficient, and economical. This technique could be applied in the papaya industry to rapidly identify the sex of papaya plants in a field-based DNA screening approach.

## 2. Results and Discussion

### 2.1. Loop-Mediated Isothermal Amplification (LAMP) Analysis for the Male-Specific Region of the Y Chromosome Using Intact Plant Material as the DNA Source

**Figure 1:**
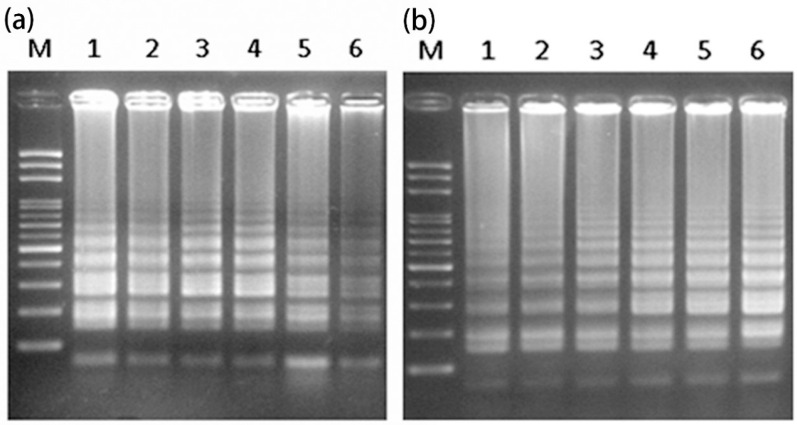
Optimization of reaction parameters: (**a**) temperature (Lanes 1–6 represent 56, 59, 62, 65, 68, and 71 °C, respectively) and (**b**) time (Lanes 1–6 represent 30, 40, 50, 60, 70, and 80 min, respectively) for LAMP analysis using extracted DNA from the leaf of a hermaphrodite plant. LAMP analysis, loop-mediated isothermal amplification (LAMP) analysis; M, mark.

To reduce the cost and time needed for sex determination of papaya, LAMP analysis was attempted using intact plant materials as a DNA source. The detected results of the male-specific region of the Y chromosome, using LAMP, initially yielded a small smear [16]. Therefore, a new set of six primers was designed and optimized for further detection (Figure S1, Table 1). Different reaction temperatures of 56, 59, 62, 65, 68, and 71 °C were examined to test the efficiency of the LAMP amplification. The results indicated that all experiment temperatures gave successful amplification. Considering the relative concentration and clarity of the LAMP products, 65 °C was found to be the optimum temperature (Figure 1a and Figure S2). Using this reaction temperature, different reaction times of 30, 40, 50, 60, 70 and 80 min were examined. Based on the relative concentration of the LAMP products—and to reduce the reaction time—a reaction time of 60 min was selected (Figure 1b and Figure S2).

**Figure 2:**
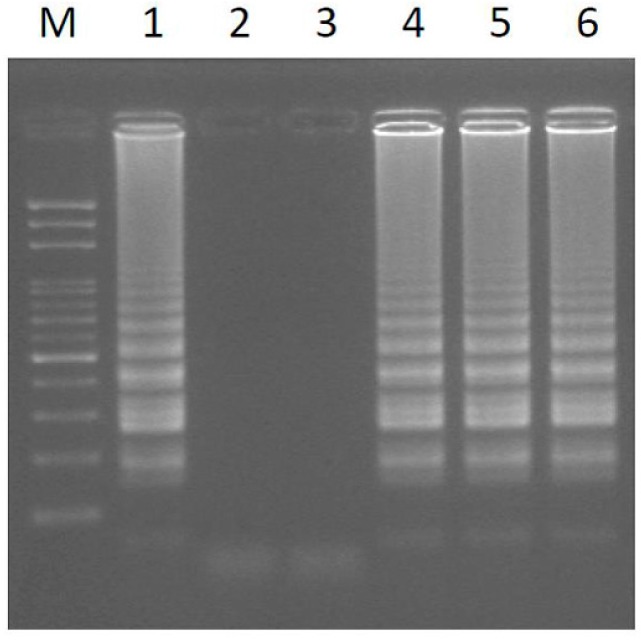
LAMP amplification was conducted using extracted DNA from the leaves of papaya under optimized reaction conditions. Lanes 1–6 represent six sex-characterized papaya plants (see Table 2). M, mark.

Using the optimized reaction temperature and time, the newly designed primer set gave highly reproducible and efficient LAMP amplification, using extracted DNA as templates for detecting the sex of six characterized samples of papaya. All of the male and hermaphrodite plants show LAMP-amplified products (Figure 2). The results show agreement with the sex types of the six samples (Table 2). Compared to a previous LAMP assay for sex determination of papaya [16], the DNA pattern of LAMP amplification is sharper and clearer.

**Figure 3:**
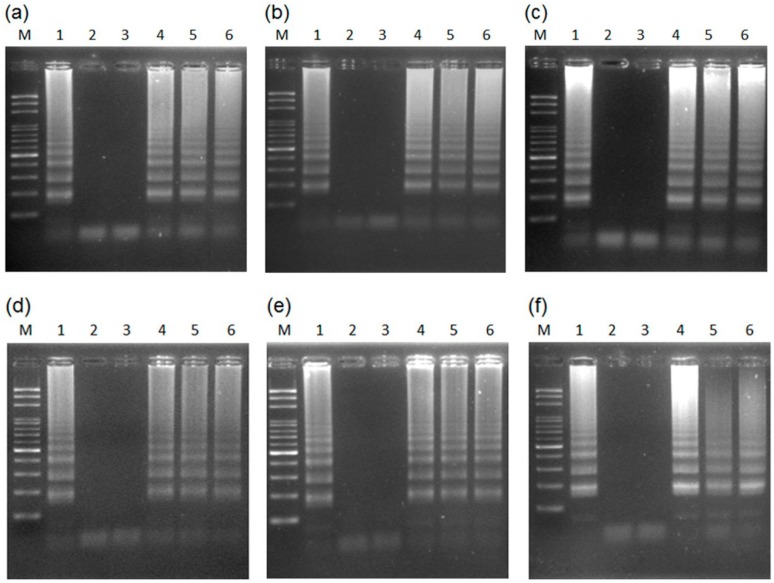
LAMP amplification can be performed using different intact leaf disk cuttings with different sizes, as a DNA source, and obtained using: (**a**) white (1–10 µL); (**b**) yellow (20–200 µL); and (**c**) blue (100–1000 µL) pipette tips, as well as distinct leaf disks in different reaction volumes of (**d**) 50 µL; (**e**) 25 µL; and (**f**) 12.5 µL, with the same concentration of the reaction mixture. Lanes 1–6 represent six sex-characterized papaya plants (see Table 2). M, mark.

Next, to evaluate the sensitivity and efficacy of the different sizes of intact plant materials as a template DNA source, samples were taken using different pipette tips: white (1–10 µL); yellow (20–200 µL); and blue (100–1000 µL) tips [39]. The small leaf disk was pipetted into a reaction tube containing reaction buffer without *Bst* DNA polymerase [40]. The result shows that the LAMP amplification can be performed using small intact leaf disks as a DNA source and is compatible with different cutting sizes (Figure 3a–c). Using the same concentrations in the reaction mixture, the assay was done using different leaf disks and was compatible with different reaction volumes of 50, 25, and 12.5 µL (Figure 3d–f). To measure the sensitivity of the LAMP papaya assay, the purified genomic DNA was diluted in 10-fold increments (10^−0^ to 10^−5^) using the standard LAMP reaction. The standard LAMP reaction was conducted in 10 ng of genomic DNA, and the results showed that the limit of amplification is 10^−2^ dilutions (0.1 ng) of genomic DNA extracted from papaya leaves (Figure S3). This method can largely reduce the cost of papaya sex determination by LAMP assay and can be performed easily and rapidly in the field.

Male and hermaphrodite-specific DNA markers have been applied for sex determination using various DNA fingerprinting techniques, such as RAPD [3,7,8], AFLP [9], and the LAMP assay [41]. Although the markers cannot separate males from hermaphrodites, they are still very useful in the papaya industry because the sex types used in commercial papaya production are either female or hermaphrodite [2]. Because the LAMP amplification is very efficient, it should be conducted carefully to avoid contamination [41]. Using intact plant material as a template, the DNA source can reduce the risk of contamination-which is high-when using plant tissues homogenized with liquid nitrogen and a mortar and pestle for general DNA extraction [42].

Plant samples usually contain components that interfere with PCR, such as phenolic compounds. This makes DNA analysis by direct PCR methods very difficult, so an additional step is usually required to remove the compounds [25,43]. Direct PCR amplification can be overcome by using a quick and easy dilution protocol, coupled with a unique DNA polymerase that has a double-stranded DNA binding domain [44]. The results showed that it is very efficient to use the small leaf disks for the sex determination of papaya.

### 2.2. Transferability of Papaya Varieties and Error Rate of Sex Determination for LAMP without Prior DNA Purification

**Figure 4:**
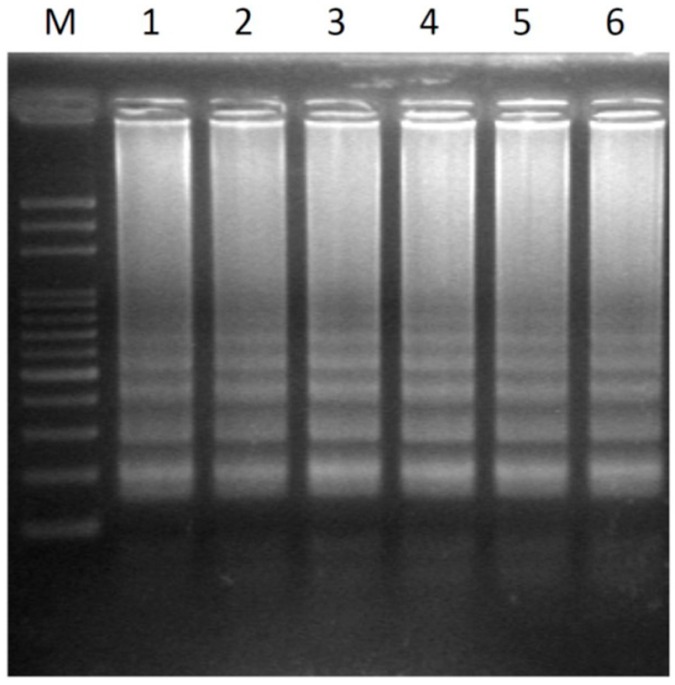
Transferability of six different papaya varieties using the LAMP assay without prior DNA purification detected by agarose gel electrophoresis. Lanes 1–6 represent hermaphrodite plants of the cultivars Tainung No.1, Tainung No.2, Red Lady, Holland, Golden, and Sunrise. M, mark.

Six hermaphrodite samples from commercial papaya varieties were tested using LAMP without prior DNA purification. The white pipette tip was used to take a leaf disk sample as a template DNA source, and the optimized conditions were used in the LAMP reaction. All of the test samples showed high-efficiency LAMP-amplification DNA patterns (Figure 4). The results showed that the detection system can be applied to determine the sex type of all of these papaya varieties.

**Figure 5:**
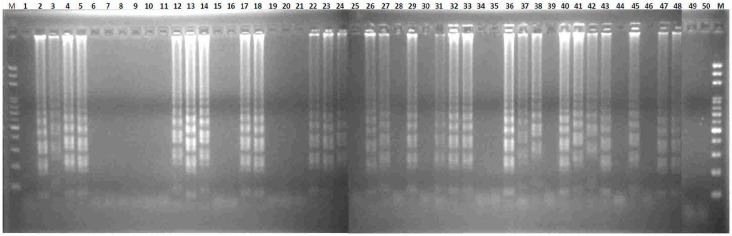
Validation of the LAMP assay without prior DNA purification. Fifty sex-uncharacterized plants of the “Red Lady” variety were used for the LAMP assay without prior DNA purification and detected by agarose gel electrophoresis. M, mark.

**Figure 6:**
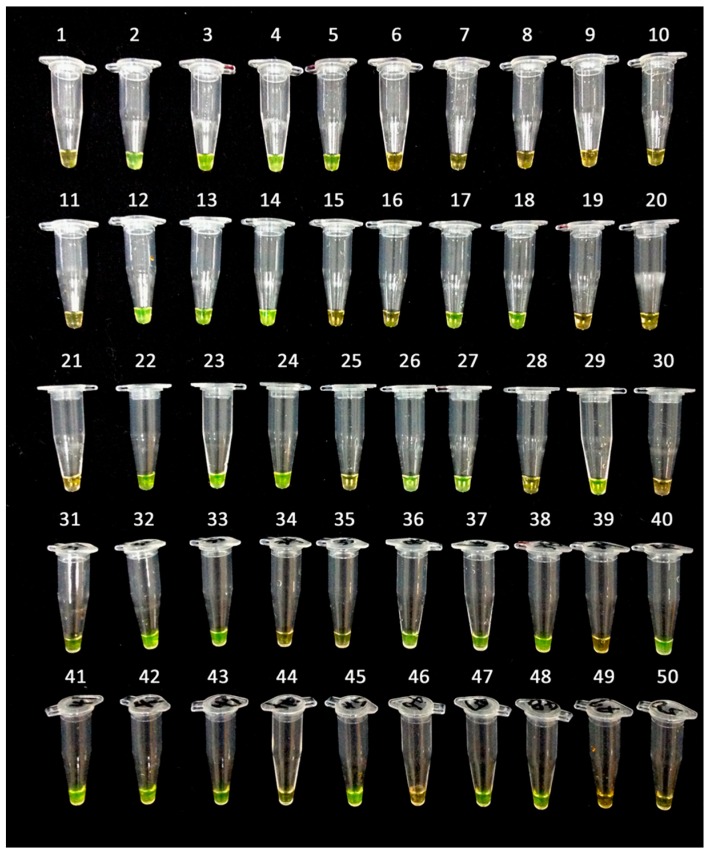
Validation of the LAMP assay without prior DNA purification. Fifty uncharacterized papaya varieties were used for the LAMP assay without prior DNA purification and detected by dying SYBR Green, under exposure, with a black background. The tubes with bright green mixtures indicate hermaphrodite plants.

Fifty uncharacterized papaya seedlings (either hermaphrodite or female plants) were also used to test the error rate. After LAMP amplification, amplified products were separated by agarose gel electrophoresis (Figure 5). Each hermaphrodite plant showed LAMP amplification. The results were also supported by the direct inspection of the reaction mixture after dying with SYBR Green against a black background (Figure 6). The hermaphrodite plants, predicted by LAMP testing, agreed perfectly with the observed sex type—when the plants eventually flowered in the field (data not shown). The test was 100% reliable (Table S1). Thus, the method is not only highly efficient and sensitive, but also has a very low error rate. It, therefore, has the potential application for sex determination of papaya in the field setting.

This technique could largely reduce the cost and time since DNA extraction is usually a limiting step for molecular assays [32]. Different reaction volumes (12.5, 25 and 50 µL) and different sizes of intact leaf disks were also all compatible with the LAMP assay (Figure 3), which helps simplify the application of the technique.

### 2.3. Mechanism of LAMP Assay without Prior DNA Purification

There are two possible explanations for the success of the direct LAMP assay without prior DNA purification. First, the LAMP amplification has 100 to 1000 times higher amplification efficiency than PCR amplification [45,46]. Therefore, the LAMP assay is effective when using the trace genomic DNA released from a small leaf disk cutting as DNA templates, and the concentration of inhibitors released from the disk is too low to interfere with *Bst* DNA polymerase. Second, *Bst* DNA polymerases are more tolerant of inhibitors than *Taq* DNA polymerase.

**Figure 7:**
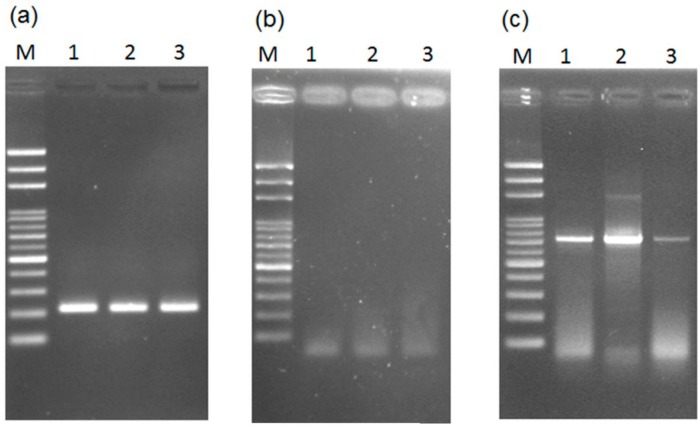

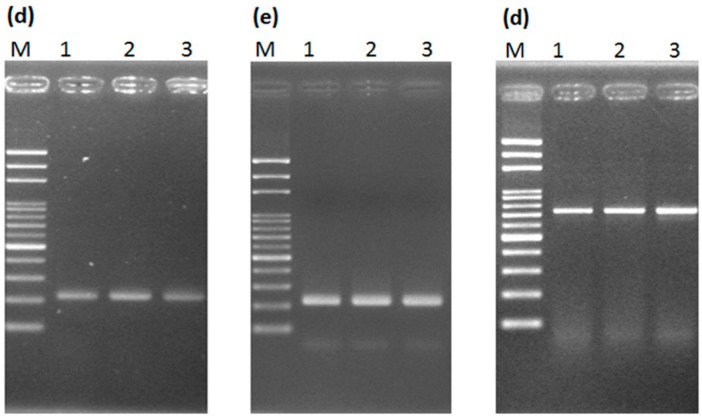
Results of testing the necessity of prior DNA purification for PCR reaction with (**a**–**c**) general and (**d**–**f**) high-efficiency DNA polymerase. Detection of the male-specific region of the Y chromosome from papaya (**a**) with prior DNA purification and (**b**) without prior DNA purification; (**c**) detection of the internal transcribed spacer (ITS) in papaya without prior DNA purification; male-specific region detection (**d**) with prior DNA purification and (**e**) without prior purification; (**f**) ITS detection without prior DNA purification. Lanes 1–3 represent hermaphrodite plants of the varieties “Tainung No. 1”, “Tainung No. 2”, and “Red Lady”. M, mark.

The PCR assay was done with and without prior DNA purification using the *Taq* DNA polymerase. The expected PCR products for the male-specific region of the Y chromosome can be amplified by the general *Taq* DNA polymerase with prior DNA purification for papaya leaves (Figure 7a)—but not when using a small leaf disk as a DNA source (Figure 7b). The results agree with most studies, showing that PCR amplification cannot be done without prior DNA purification of the plant materials. This is usually attributed to the several inhibitors of PCR DNA polymerase in plants; such as chlorophyll, polysaccharides, starches, polyphenols, or other secondary metabolites [21,37].

In addition to the inhibitors of DNA polymerase, another issue is that insufficient DNA is released from the small leaf disk cuttings for amplification by general *Taq* DNA polymerase. To verify this, another multiple copy gene, the ITS region of rDNA, was targeted for the PCR assay using a small leaf disk as a DNA source. The results showed the expected PCR products (Figure 7c), which was confirmed by sequencing (data not shown). The results confirm that insufficient DNA was released from the disks.

Next, we predicted that conventional PCR analysis could be done without prior DNA purification based on the combination of a leaf disk and high-efficiency DNA polymerase. To verify this, high-efficiency Advantage 2 DNA polymerase [40] was used for PCR assay with a small leaf disk as a DNA source to separately amplify the male-specific region and the ITS region. The expected PCR products for both regions were confirmed by sequencing (data not shown). The results showed that both regions can be efficiently amplified in this way (Figure 7d–f).

In summary, the amplification efficiency of DNA polymerase is a limiting factor for PCR amplification when using a small leaf disk as a DNA source. Based on the results, the mechanism of the LAMP assay was determined. We concluded that the level of inhibitors released from the disk are too low to interfere with *Bst* DNA polymerase for LAMP amplification, and trace DNA released from the disk cutting is sufficient for the detection of a single copy gene. The protocol of obtaining DNA for genotyping could be applied for the PCR assay by using high-efficiency DNA polymerase. This strategy could offer great potential to develop a quick and economical genotyping method for other plants in the future.

## 3. Materials and Methods

### 3.1. Plant Materials

Six sex-characterized papaya plants were examined, including two hermaphrodites, two females, and two males (Table 2). The transferability of different papaya varieties was tested using commercial papaya samples from the hermaphrodite plants (Table 3). In addition, 50 uncharacterized papaya seeds of the “Red Lady” variety were purchased from Known-You Seed Co., Ltd., Kaohsiung, Taiwan. These seeds were also sown and cultivated to validate the sex determination technique. The plants were cultivated at Kaohsiung District Agricultural Research and Extension (KDARES), Pingtung, Taiwan.

### 3.2. Design of the Primers for the Male-Specific Region of Y Chromosome for LAMP Assay

According to Notomi et al. [12], a set of six primers was designed using Primer Explorer V5 software [47] and used for the LAMP analysis derived from the male-specific region of the Y chromosome (Figure S1). The set of primers includes two outer primers (F3 and B3), two inner primers (F2 and B2), and two loop-forming primers (O-FIP and O-BIP), which recognize six different regions in the target sequence. The F2 and F1c primers connect together as the loop primer FIP, while the B2 and B1c primers connect together as the loop primer BIP (Table 1).

### 3.3. DNA Extraction and Optimization of the LAMP Assay

DNA was extracted from fresh papaya leaves using a Qiagen DNeasy Plant Mini Kit (Valencia, CA, USA) and was measured by a NanoDrop ND-1000 spectrophotometer (Thermo Fisher Scientific Inc., Waltham, MA, USA). The standard LAMP reaction was conducted in a 25 μL mixture containing 1× betaine buffer mix, 1.4 mM dNTPs, 0.2 µM F3 primers, 0.2 µM B3 primers, 1 µM FIP primers, 1 µM BIP primers, 1 µM LF primers, 1 µM LB primers, and 10 ng of genomic DNA (Table S2). Various reaction temperatures and times in the LAMP analysis were examined to optimize the reaction conditions for sex determination. Reaction temperatures of 56, 59, 62, 65, 68, and 71 °C were tested, and the reaction times were 30, 40, 50, 60, 70, and 80 min. Finally, 3 μl of LAMP products were separated on 2.0% agarose gel. The amplified products were visible under ultraviolet light after ethidium bromide (EtBr) staining.

### 3.4. LAMP Assay without Prior DNA Purification and Its Compatibility

The LAMP reaction was conducted in a 25 μL mixture with the same concentrations, along with one small piece of leaf disk cutting obtained using a 100–1000 µL pipette tip. The process of the LAMP assay, without DNA purification, is shown in Figure S4. The mixture with the intact leaf disk was incubated at 95 °C for 10 min in a heating block, and then 8 U *Bst* DNA polymerase was added (New England Biolabs, Beverly, MA, USA). The mixture was incubated at 65 °C for one hour using the heating block, followed by heating at 80 °C for 10 min to terminate the reaction. Finally, 3 μL of LAMP products were separated on 2.0% agarose gel. The amplified products were visible under ultraviolet light after EtBr staining. To test the compatibility of the assay, different sample sizes were examined using cuttings obtained with white (1–10 µL), yellow (20–200 µL), and blue (100–1000 µL) tips—using the same LAMP mixture concentrations.

### 3.5. PCR Amplification with and without Prior DNA Purification by Two Different DNA Polymerases for Single/Multiple Genes

PCR was done with and without prior DNA purification for the male-specific region of the Y chromosome (single copy gene) [18,19] and for the internal transcribed spacer (ITS) region of ribosomal DNA (rDNA) (multiple copy gene) [39] by using general *Taq* DNA polymerase. The procedure was done using the following: 25 µL of 2× Red Mix DNA Polymerase Mastermix (RBC Bioscience, New Taipei, Taiwan) containing 20 mM KCl, 3 mM MgCl_2_, 40 mM Tris-HCl (pH 8.8), 0.2% Tween 20, 0.4 mM dNTP mix, and 0.1 U/µL RBC *Taq* DNA polymerase; primers F3 and B3 (0.4 µM each) for the male-specific region of the Y chromosome or universal primers (0.4 µM each) for the ITS in higher plants designed by Tsai et al. [48]. A sample of 10 ng of genomic DNA or a small leaf disk cutting by a yellow tip was used for PCR amplification of the male-specific region of the Y chromosome; whereas, only the cutting was used for the PCR amplification of the ITS region (Table S3).

The PCR without prior DNA purification was also done for these two regions using high-efficiency Advantage 2 DNA polymerase [40] with the following: a 25-µL mixture containing 40 mM Tricine-KOH (pH 8.7), 15 mM KOAc, 3.5 mM Mg(OAc)_2_, 3.75 µg/mL BSA, 0.005% Tween 20, 0.005% Nonidet-P40, four dNTPs (0.2 mM each), and primers F3 and B3 (0.4 µM each) for the male-specific region of the Y chromosome (Figure S1); or, universal primers (0.4 µM each) for the ITS region, 1.25 units of Advantage 2 DNA polymerase (Clontech Laboratories, Inc., Mountain View, CA, USA), and a small leaf disk cutting by a yellow tip as DNA source (Table S3).

All of the above PCR reactions were performed on a thermocycler (Biometra, Göttingen, Germany) under the following conditions: 94 °C for 5 min followed by 35 cycles of denaturation at 94 °C for 15 s, annealing at 53 °C for 15 s, extension at 72 °C for 20 s, and a final extension at 72° C for 5 min. The PCR products were visualized on 2% agarose gel. A product of the expected size was amplified from each of the samples.

### 3.6. DNA Sequencing

The amplified products were purified using Qiagen columns (Valencia, Orlando, FL, USA). The PCR products were cloned into the pGEM-T Easy Vector (Promega, Madison, WI, USA), and five independent clones were sequenced. The cloned DNA was sequenced using the dideoxy chain termination method with an ABI3730 automated sequencer and the BigDye™ Terminator Cycle Sequencing Ready Reaction Kit (PE Biosystems, Foster City, CA, USA). The sequencing reactions were performed as recommended by the manufacturers.

## 4. Conclusions

A more rapid and efficient method for detecting the male-hermaphrodite-specific marker was developed to examine papaya sex type based on LAMP assays without prior DNA purification. The results of sensitivity and the efficacy tests showed that the LAMP amplification can be performed using a small, intact leaf disk as a DNA source with a small reaction mixture and direct inspection of the reaction mixture dyed by SYBR Green. The results showed that the method is easy, efficient, and inexpensive. Different papaya varieties and a large number of papaya seedlings were used to test the transferability and the error rate, and the results showed a 100% success rate. This is the first report on using intact plant materials without prior DNA purification for LAMP assays to examine the papaya sex types. The technique could be helpful to the papaya industry for rapid and effective sex determination. It was also verified that using high-efficiency DNA polymerase is essential for the DNA amplification using this method.

## Figures and Tables

**Table 1:** A set of primers for sex determination using loop-mediated isothermal amplification (LAMP) analysis.

Primer Name	Sequence
papaya-F3	5′-GTGGCATTAATGCAACGC-3′
papaya-B3	5′-TGTCACCATGAGCACTAG-3′
papaya-FIP	5′-CAGAGGAAGAGTGGGTGTTTTGCGGGTCCTACGAACCTAG-3′
papaya-BIP	5′-GCTTGCCGAACATAGAGGCTTGAGCACGATCCACGAGAT-3′
papaya-LF	5′-GTGGTGTTGTAGGCATCAAATGTT-3′
papaya-LB	5′-TCTCCCCTCACACCCAATCC-3′

**Table 2:** Six sex-characterized papaya plants used for the characterization of LAMP analysis.

Papaya Sample No.	Varieties Name	Sex Type
1	Line Ph13047-1	hermaphrodite
2	Inbred line Y5043	Females
3	Inbred line Y5043	Females
4	Inbred line Y5043	Males
5	Line Ph13047-2	hermaphrodite
6	Inbred line Y5043	Males

**Table 3:** Plant materials used for examining the transferability of LAMP analysis without prior DNA purification.

Papaya Sample No.	Varieties Name	Source
1	Tainung No. 1	Taiwan
2	Tainung No. 2	Taiwan
3	Red Lady	Taiwan
4	Holland	Thailand
5	Golden	Brazil
6	Sunrise	Brazil

## Supplementary Materials

Supplementary materials can be found at www.mdpi.com/1422-0067/17/10/1630/s1.

## References

[1] Dallwitz M.J. (1980). A general system for coding taxonomic descriptions. Taxon.

[2] Chan T.C., Yen C.R., Chang L.S., Hsiao C.H., Ko T.S. (2003). All hermaphrodite progeny are derived by self-pollinating the sunrise papaya mutant. Plant Breed..

[3] Parasnis A.S., Gupta V.S., Tamhankar S.A., Ranjekar P.K. (2000). A highly reliable sex diagnostic PCR assay for mass screening of papaya seedlings. Mol. Breed..

[4] Tsai C.C., Shih H.C., Wang H.V., Lin Y.S., Chang C.H., Chiang Y.C., Chou C.H. (2015). RNA-seq SSRs of moth orchid and screening for molecular markers across genus *Phalaenopsis* (Orchidaceae). PLoS ONE.

[5] Tsai C.C., Chen Y.K.H., Chen C.H., Weng I.S., Tsai C.M., Lee S.R., Lin Y.S., Chiang Y.C. (2013). Cultivar identification and genetic relationship of mango (*Mangifera indica*) in Taiwan using 37 SSR markers. Sci. Hortic..

[6] Lai J.M., Tsai C.C., Yen C.R., Ko Y.Z., Chen S.R., Weng I.S., Lin Y.S., Chiang Y.C. (2015). Molecular characterization of twenty polymorphic microsatellite markers in the polyploid fruit tree species *Syzygium samarangense* (Myrtaceae). Genet. Mol. Res..

[7] Deputy J.C., Ming R., Ma H., Liu Z., Fitch M., Wang M., Manshardt R., Stiles J.L. (2002). Molecular markers for sex determination in papaya (*Carica papaya* L.). Theor. Appl. Genet..

[8] Urasaki N., Tokumoto M., Tarora K., Ban Y., Kayano T., Tanaka H., Oku H., Chinen I., Terauchi R. (2002). A male and hermaphrodite specific RAPD marker for papaya (*Carica papaya* L.). Theor. Appl. Genet..

[9] Guo X., Zhang Y., Liu Z.Y. (2009). Development of sex linked AFLP-derived SCAR markers in *Cairca papaya*. Sci. Agric. Sin..

[10] Sobir S.S., Pandia E.C. (2008). Development of SCAR marker for detection of sex expression in papaya (*Carica papaya* L.) from several genetic backgrounds. Bul. Agron..

[11] Rigano L.A., Marano M.R., Castagnaro A.P., Do Amaral A.M., Vojnov A.A. (2010). Rapid and sensitive detection of Citrus Bacterial Canker by loop-mediated isothermal amplification combined with simple visual evaluation methods. BMC Microbiol..

[12] Notomi T., Okayama H., Masubuchi H., Yonekawa T., Watanabe K., Amino N., Hase T. (2000). Loop-mediated isothermal amplification of DNA. Nucleic Acids Res..

[13] Fukuta S., Ohishi K., Yoshida K., Mizukami Y., Ishida A., Kanbe M. (2004). Development of immunocapture reverse transcription loop-mediated isothermal amplification for the detection of tomato spotted wilt virus from chrysanthemum. J. Virol. Methods.

[14] Buates S., Bantuchai S., Sattabongkot J., Han E.T., Tsuboi T., Udomsangpetch R., Sirichaisinthop J., Tan-ariya P. (2010). Development of a reverse transcription-loop-mediated isothermal amplification (RT-LAMP) for clinical detection of *Plasmodium falciparum* gametocytes. Parasitol. Int..

[15] Huang C.H., Lai G.H., Lee M.S., Lin W., Lien Y.Y., Hsueh S.C., Kao J.Y., Chang W.T., Lu T.C., Lin W.N. (2010). Development and evaluation of a loop-mediated isothermal amplification assay for rapid detection of chicken anaemia virus. J. Appl. Microbiol..

[16] Hsu T.H., Gwo J.C., Lin K.H. (2012). Rapid sex identification of papaya (*Carica papaya*) using multiplex loop-mediated isothermal amplification (mLAMP). Planta.

[17] Chan K.W., Liu P.C., Yang W.C., Kuo J., Chang C.L., Wang C.Y. (2012). A novel loop-mediated isothermal amplification approach for sex identification of Columbidae birds. Theriogenology.

[18] Liu Z., Moore P.H., Ma H., Ackerman C.M., Ragiba M., Yu Q., Pearl H.M., Kim M.S., Charlton J.W., Stiles J.I. (2004). A primitive Y chromosome in papaya marks incipient sex chromosome evolution. Nature.

[19] Yu Q., Hou S., Hobza R., Feltus F.A., Wang X., Jin W., Skelton R.S., Blas A., Lemke C., Saw J.H. (2007). Chromosomal location and gene paucity of the male specific region on papaya Y chromosome. Mol. Genet. Genom..

[20] Porebski S., Bailey L.G., Baum B.R. (1997). Modification of a CTAB DNA extraction protocol for plants containing high polysaccharide and polyphenol components. Plant Mol. Biol. Rep..

[21] Friar E.A. (2005). Isolation of DNA from plants with large amounts of secondary metabolites. Methods Enzymol..

[22] Katterman F.R., Shattuck V.I. (1983). An effective method of DNA isolation from the mature leaves of *Gossypium* species that contain large amounts of phenolic terpenoids and tannins. Prep. Biochem..

[23] Pandey R.N., Adams R.P., Flournoy L.E. (1996). Inhibition of random amplified polymorphic DNAs (RAPDs) by plant polysaccharides. Plant Mol. Biol. Rep..

[24] Stange C., Prehn D., Arce-Johnson P. (1998). Isolation of *Pinus radiata* genomic DNA suitable for RAPD analysis. Plant Mol. Biol. Rep..

[25] Kim C.S., Lee C.H., Shin J.S., Chung Y.S., Hyung N.I. (1997). A simple and rapid method for isolation of high quality genomic DNA from fruit trees and conifers using PVP. Nucleic Acids Res..

[26] Kaufman B., Richards S., Dierig D.A. (1999). DNA isolation method for high polysaccharide *Lesquerella* species. Ind. Crop. Prod..

[27] Michiels A., van den Ende W., Tucker M., van Riet L., van Laere A. (2003). Extraction of high-quality genomic DNA from latex-containing plants. Anal. Biochem..

[28] Echevarría-Machado I., Sánchez-Cach L.A., Hernández-Zepeda C., Rivera-Madrid R., Moreno-Valenzuela O.A. (2005). A simple and efficient method for isolation of DNA in high mucilaginous plant tissues. Mol. Biotechnol..

[29] Moyo M., Amoo S.O., Bairu M.W., Finnie J.F., van Staden J. (2008). Optimising DNA isolation for medicinal plants. S. Afr. J. Bot..

[30] Nagori R., Sharma P., Habibi N., Purohit S.D. (2014). An efficient genomic DNA extraction protocol for molecular analysis in *Annona reticulata*. Natl. Acad. Sci. Lett..

[31] Fang G., Hammar S., Grumet R. (1992). A quick and inexpensive method for removing polysaccharides from plant genomic DNA. Biotechniques.

[32] Das S., Tiwari K.L., Sen S., Singh A. (2012). Rapid one step DNA extraction method from (*Gastrimargus musicus*) through formaldehyde. J. Pharm. Biomed. Sci..

[33] Grevelding C.G., Kampkötter A., Hollmann M., Schäfer U., Kunz W. (1996). Direct PCR on fruitflies and blood flukes without prior DNA isolation. Nucleic Acids Res..

[34] Bu Y., Huang H., Zhou G. (2008). Direct polymerase chain reaction (PCR) from human whole blood and filter-paper-dried blood by using a PCR buffer with a higher pH. Anal. Biochem..

[35] Li H., Xu H., Zhao C., Sulaiman Y., Wu C. (2011). A PCR amplification method without DNA extraction. Electrophoresis.

[36] Sharma R., Virdi A.S., Singh P. (2012). A novel method for whole blood PCR without pretreatment. Gene.

[37] Yang Y.G., Kim J.Y., Soh M.S., Kim D.S. (2007). A simple and rapid gene amplification from Arabidopsis leaves using AnyDirect system. J. Biochem. Mol. Biol..

[38] Chang W.H., Yang S.Y., Lin C.L., Wang C.H., Li P.C., Chen T.Y., Jan F.J., Lee G.B. (2013). Detection of viruses directly from the fresh leaves of a *Phalaenopsis* orchid using a microfluidic system. Nanomedicine.

[39] Bellstedt D.U., Pirie M.D., Visser J.C., de Villiers M.J., Gehrke B. (2010). A rapid and inexpensive method for the direct PCR amplification of DNA from plants. Am. J. Bot..

[40] Ming R., Hou S., Feng Y., Yu Q., Dionne-Laporte A., Saw J.H., Senin P., Wang W., Ly B.V., Lewis K.L.T. (2008). The draft genome of the transgenic tropical fruit tree papaya (*Carica papaya* Linnaeus). Nature.

[41] Rocha A.J., de Souza Miranda R., da Silva Cunha R.M. (2014). Assessment of DNA polymerases in microsatellite amplification assays through PowerPlex^®^ 16 BIO System. Biochem. Biotechnol. Rep..

[42] Rohland N., Hofreiter M. (2007). Ancient DNA extraction from bones and teeth. Nat. Protoc..

[43] John M.E. (1992). An efficient method for isolation of RNA and DNA from plants containing polyphenolics. Nucleic Acids Res..

[44] Chum P.Y., Haimes J.D., André C.P., Kuusisto P.K., Kelley M.L. (2014). Genotyping of plant and animal samples without prior DNA purification. J. Vis. Exp..

[45] Kong Q.M., Lu S.H., Tong Q.B., Lou D., Chen R., Zheng B., Kumagai T., Wen L.Y., Ohta N., Zhou X.N. (2012). Loop-mediated isothermal amplification (LAMP): Early detection of *Toxoplasma gondii* infection in mice. Parasites Vectors.

[46] Ming R., Yu Q.Y., Moore P.H. (2007). Sex determination in papaya. Semin. Cell Dev. Biol..

[47] Eiken Chemical Co., Ltd. (2016). Primer Explorer Version 4 Software. http://primerexplorer.jp/v5_manual/index.html.

[48] Tsai C.C., Huang S.C., Chen C.H., Tseng Y.H., Huang P.L., Tsai S.H., Chou C.H. (2003). Genetic relationship of *Rhododendron* (Ericaceae) in Taiwan based on the sequence of the internal transcribed spacer of ribosomal DNA. J. Hortic. Sci. Biotechnol..

