# Supplementary material for: Direct LAMP Assay without Prior DNA Purification for Sex Determination of Papaya
Source: Int J Mol Sci. 2016 Sep 24;17(10):1630. doi: 10.3390/ijms17101630 (PMC5085663; doi:10.3390/ijms17101630)
Supplement: Supplementary file 1 [file ijms-17-01630-s001.pdf]

Int. J. Mol. Sci. **2016**, *17*, 1630; doi:10.3390/ijms17101630

Supplementary Material: Direct LAMP Assay without Prior DNA Purification for Sex Determination of Papaya

Chi-Chu Tsai, Huei-Chuan Shih, Ya-Zhu Ko, Ren-Huang Wang, Shu-Ju Li and Yu-Chung Chiang

```
                                        F3                                F2
AY428939.1   ATTCCCCCCA TGAAGTGGCA TTAATGCAAC GCATGTTAAA AACCTGCGGG TCCTACGAAC
AY861344.1   .......... .......... .......... .......... .......... ..........
AY849325.1   .......... ..C....... .......... .......... .......... ..........
AY428938.1   .......... ..C....... .......... .......... .......... ..........
FJ011100.1   .......... .......... .......... .......... .......... ..........
FJ011101.1   .......... ..C....... .......... .......... .......... ..........
FJ011102.1   .......... ..C....... .......... .......... .......... ..........
FJ011103.1   .......... ..C....... .......... .......... .......... ..........
                              LF                          F1c
AY428939.1   CTAGAACATT TGATGCCTAC AACACCACTT ACAAAACACC CACTCTTCCT CTGCTAATTC
AY861344.1   .......... .......... .......... .......... .......... ..........
AY849325.1   .......... .......... .......... .......... .......... ..........
AY428938.1   .......... .......... .......... .......... .......... ..........
FJ011100.1   .......... .......... .......... .......... .......... ..........
FJ011101.1   .......... .......... .......... .......... .......... ..........
FJ011102.1   .......... .......... .......... .......... .......... ..........
FJ011103.1   .......... .......... .......... .......... .......... ..........
                                         B1c
AY428939.1   TTGTAATTGT CAGCGTGCTT GCCGAACATA GAGGCTTTCG GCCTCACTAA CCTTTCTCCC
AY861344.1   .......... .......... .......... .......... .......... ..........
AY849325.1   .......... .......... .......... .......... .......... ..........
AY428938.1   .......... .......... .......... .......... .......... ..........
FJ011100.1   .......... .......... .......... .......... .......... ..........
FJ011101.1   .......... .......... .......... .......... .......... ..........
FJ011102.1   .......... .......... .......... .......... .......... ..........
FJ011103.1   .......... .......... .......... .......... .......... ..........
                  LB                      B2                     B3
AY428939.1   CTCACACCCA AATCCCATAA ATCTCGTGGA TCGTGCTCCT AGTGCTCATG GTGACACCCG
AY861344.1   .......... .......... .......... .......... .......... ..........
AY849325.1   .......... .......... .......... .......... .......... ..........
AY428938.1   .......... .......... .......... .......... .......... ..........
FJ011100.1   .......... .......... .......... .......... .......... ..........
FJ011101.1   .......... .......... .......... .......... .......... ..........
FJ011102.1   .......... .......... .......... .......... .......... ..........
FJ011103.1   .......... .......... .......... .......... .......... ..........
```

Figure S1. Alignment of the DNA sequence of the loop-mediated isothermal amplification (LAMP) primer-recognized regions for eight isolates of the male-specific region of the Y chromosome in papaya. Nucleotide sequences differing from that of AY428939.1 are shown as lower case letters, and identical nucleotides are shown as periods. The sequences of primer sites are boxed. The eight isolates are from Taiwan (AY861344.1 and AY849325.1), USA (AY428939.1 and AY428938.1), and Colombia (FJ011100.1, FJ011101.1, FJ011102.1 and FJ011103.1) and were obtained from GenBank.

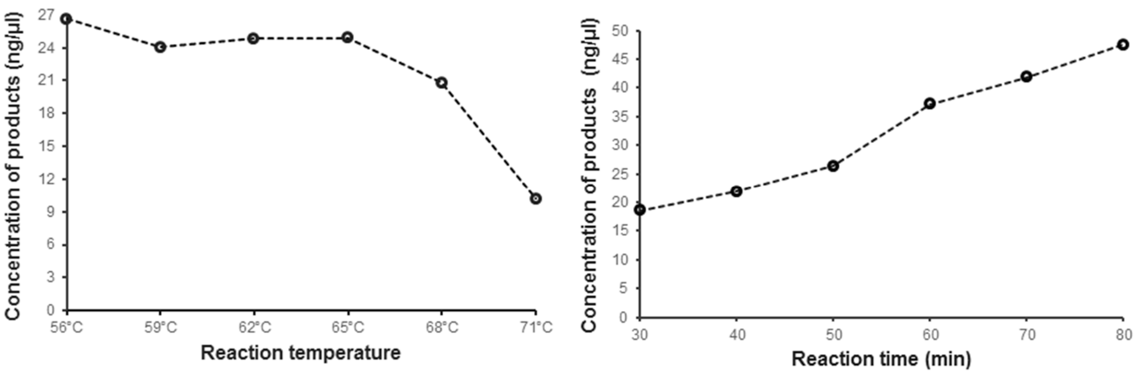

Figure S2. The relative concentration of the LAMP amplification products in the optimization test of LAMP reactions for different reaction temperatures and reaction times.

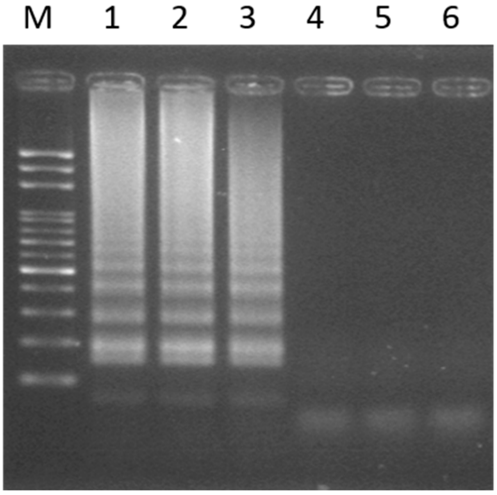

Figure S3. Amplification sensitivity test of 10-fold serial dilutions (10^{-0} to 10^{-5}) of papaya genomic DNA. The standard LAMP reaction was conducted in 10 ng of genomic DNA. The number 1–6 indicated a series of diluted DNA. Lane 1: 10^{-0}, Lane 2: 10^{-1}, Lane 3: 10^{-2}, Lane 4: 10^{-3}, Lane 5: 10^{-4}, and Lane 6: 10^{-5}. The standard 25 µL mixture containing 1× betaine buffer mix, 1.4 mM dNTPs, 0.2 µM F3 primers, 0.2 µM B3 primers, 1 µM FIP primers, 1 µM BIP primers, 1 µM LF primers, 1 µM LB primers, and genomic DNA.

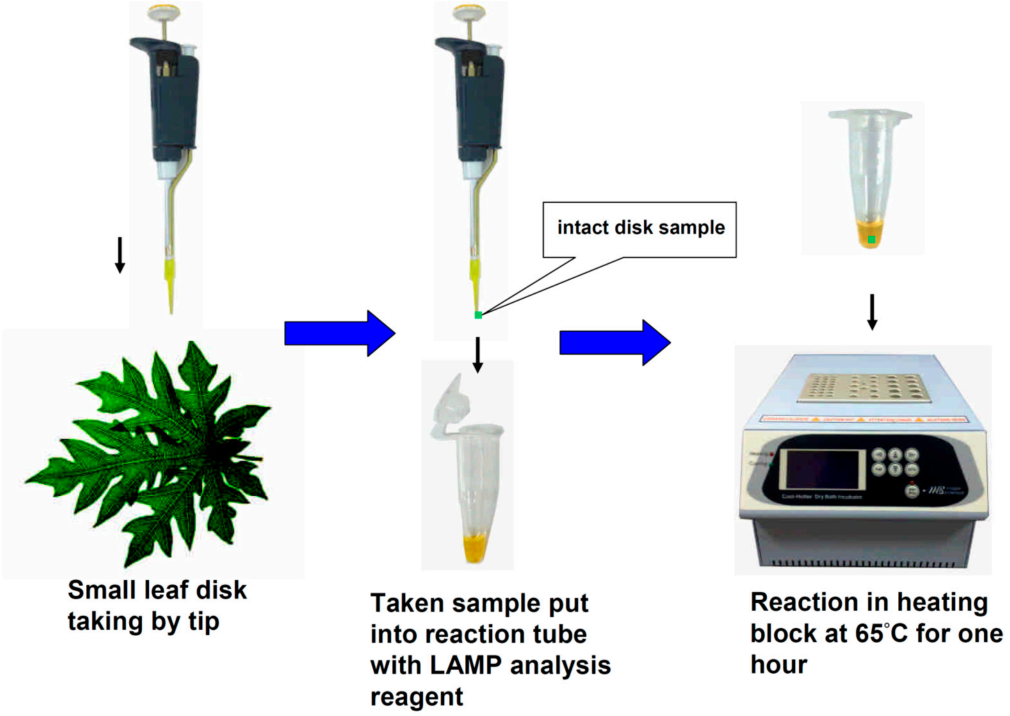

Figure S4. The flow chart of the novel direct LAMP protocol for the sex determination of papaya in the study. The guideline protocol is shown as follows: (1) The total volume of 25 μL LAMP reaction mixture with two exceptions of *Bst* DNA polymerase and small leaf disk was prepared and loaded into reaction tube; (2) small leaf disk (0.05 mm in diameter) was cut from a piece of papaya leaf using a common pipette with yellow tip; (3) the cut small leaf disk was put into the reaction tube by pipetting for several times; (4) after sealing, the reaction tube was incubated at 95 °C for 10 min; (5) after adding *Bst* DNA polymerase, the reaction tube was incubated at 65 °C for 1 h.

Table S1. Results of the success rates for the sex type detected by the LAMP and validated by adding SYBR green dye.

Sex Type	Results of LAMP Amplification/Validated by Dying SYBR Green	
	Sampling Number	Negative Result
Hermaphrodite	28	28/28
Female	22	22/22
Total	50	50/50
Success rates	-	100%/100%

Table S2. Reagent setup of optimization test for LAMP reactions used in this study.

Component	Stock Concentration	Required Concentration	Required Volume Per Reaction (μL)
Betaine reaction buffer	2×	1×	12.5
Betaine	1.6 M	-	-
Tris buffer (pH 8.8)	40 mM	-	-
$(NH_4)_2SO_4$	20 mM	-	-
KCl	20 mM	-	-
Tween 20	0.2%	-	-
$MgSO_4$	16 mM	-	-
$MnCl_2$	1 mM	-	-
Calcine	50 uM	-	-
dNTP	10 mM	1.4 mM	3.5
F3 primer	10 μM	0.2 μM	0.5
B3 primer	10 μM	0.2 μM	0.5
FIP primer	50 μM	1 μM	0.5
BIP primer	50 μM	1 μM	0.5
LB primer	50 μM	1 μM	0.5
LF primer	50 μM	1 μM	0.5
Bst polymerase	1600 U/200 μL	8 U	1
dd H_2O	-	-	4
cDNA (Heating at 95 °C for 5 min before use)	-	-	1
Total reaction volume	-	-	25

Table S3. Reagent setup for the PCR amplification of the male-specific region of the Y chromosome and for the internal transcribed spacer (ITS) region of the ribosomal DNA (rDNA) test used in this study.

Component	Stock Concentration	Required Concentration	Required Volume Per Reaction (μL)
Red Mix DNA polymerase Mastermix	2×	1×	12.5
KCl	20 mM		
$MgCl_2$	3 mM		
Tris-HCl	40 mM		
Tween 20	0.2%		
dNTP mix	0.4 mM		
RBC *Taq* DNA polymerase	0.1 U/μL		
F3 primer	10 μM	0.4 μM	1
B3 primer	10 μM	0.4 μM	1
dd H_2O	-	-	9.5
cDNA	-	-	1
Total reaction volume	-	-	25
Advantage 2 DNA polymerase:	10×	1×	
Tricine-KOH	40 mM		2.5
KOAc	15 mM		
$Mg(OAc)_2$	3.5 mM		
BSA	3.75 μg/mL		
Tween 20	0.005%		
Nonidet-P40	0.005%		
F3 primer	10 μM	0.4 μM	1
B3 primer	10 μM	0.4 μM	1
Advantage 2 DNA polymerase	1600 U/200 μL	1.25 U	0.16
dd H_2O	-	-	20.34
cDNA	-	-	-
Total reaction volume	-	-	25
